# Identifying Candidate Circulating RNA Markers for Coronary Artery Disease by Deep RNA-Sequencing in Human Plasma

**DOI:** 10.3390/cells11203191

**Published:** 2022-10-11

**Authors:** Zoe Ward, Sebastian Schmeier, John Pearson, Vicky A Cameron, Chris M Frampton, Richard W Troughton, Rob N Doughty, A. Mark Richards, Anna P Pilbrow

**Affiliations:** 1 Christchurch Heart Institute, Department of Medicine, University of Otago—Christchurch, Christchurch 8140, New Zealand; 2 School of Natural and Computational Sciences, Massey University, Auckland 0632, New Zealand; 3 Evotec SE, Essener Bogen 7, 22419 Hamburg, Germany; 4 Biostatistics and Computational Biology Unit, University of Otago—Christchurch, Christchurch 8140, New Zealand; 5 Heart Health Research Group, University of Auckland, Auckland 1023, New Zealand; 6 Cardiovascular Research Institute, National University of Singapore, Singapore 119228, Singapore

**Keywords:** RNA-sequencing, coronary artery disease, biomarker, circulating cell-free RNA, messenger RNA, long non-coding RNA, circular RNA, plasma

## Abstract

Advances in RNA sequencing (RNA-Seq) have facilitated transcriptomic analysis of plasma for the discovery of new diagnostic and prognostic markers for disease. We aimed to develop a short-read RNA-Seq protocol to detect mRNAs, long non-coding RNAs (lncRNAs) and circular RNAs (circRNAs) in plasma for the discovery of novel markers for coronary artery disease (CAD) and heart failure (HF). Circulating cell-free RNA from 59 patients with stable CAD (half of whom developed HF within 3 years) and 30 controls was sequenced to a median depth of 108 paired reads per sample. We identified fragments from 3986 messenger RNAs (mRNAs), 164 long non-coding RNAs (lncRNAs), 405 putative novel lncRNAs and 227 circular RNAs in plasma. Circulating levels of 160 mRNAs, 10 lncRNAs and 2 putative novel lncRNAs were altered in patients compared with controls (absolute fold change >1.2, *p* < 0.01 adjusted for multiple comparisons). The most differentially abundant transcripts were enriched in mRNAs encoded by the mitochondrial genome. We did not detect any differences in the plasma RNA profile between patients who developed HF compared with those who did not. In summary, we show that mRNAs, lncRNAs and circular RNAs can be reliably detected in plasma by deep RNA-Seq. Multiple coding and non-coding transcripts were altered in association with CAD, including several mitochondrial mRNAs, which may indicate underlying myocardial ischaemia and oxidative stress. If validated, circulating levels of these transcripts could potentially be used to help identify asymptomatic individuals with established CAD prior to an acute coronary event.

## 1. Introduction

Coronary artery disease (CAD) is a major cause of death worldwide [1]. The progression of CAD varies considerably between patients, making it difficult to identify those at risk of subsequent adverse events such as myocardial infarction or heart failure (HF). The cardiac troponins and natriuretic peptides are the international gold standards for diagnosis of myocardial infarction and HF, respectively, and have utility as prognostic markers [2,3]. However, no single marker can reliably predict disease progression and adverse outcomes in all patients [4], and new approaches are needed to complement current strategies.

Advances in RNA sequencing (RNA-Seq) technologies have facilitated transcriptomic analysis of plasma for the discovery of new diagnostic and prognostic markers for disease. RNA molecules are released into the circulation from healthy, apoptotic and necrotic cells and may provide insight into the health status of solid tissues that cannot be readily biopsied [5]. Circulating RNAs, including messenger RNAs (mRNAs), long non-coding RNAs (lncRNAs, >200 nucleotides in length), circular RNAs (circRNAs, RNAs that form a continuous closed loop [6]) and microRNAs (miRNAs, ~21 nucleotides in length), may be present in plasma in membrane-bound extracellular vesicles, such as exosomes, micro-vesicles and apoptotic bodies, bound to ribonucleoprotein (RNP) complexes or high-density lipoproteins [HDLs], or circulating freely [5,7,8,9]. Many RNAs are stable in plasma (especially circRNAs, which resist degradation by ribonucleases owing to their circular structure [6]), making them excellent candidate biomarkers [5]. Circulating RNAs, particularly miRNAs, but also lncRNAs and circRNAs, have already been linked to numerous diseases, including CAD and HF [10,11,12,13,14,15]. However, RNA-Seq of plasma is technically demanding: the low amounts of RNA, partial degradation of RNA due to ribonucleases in blood and the reliance on stored samples of varying ages result in challenging technical hurdles [16]. These reasons may explain why most RNA-Seq studies in biofluids have analysed small RNAs (e.g., miRNAs), with only a handful of studies attempting RNA-Seq for analysis of larger transcripts [8,9,16,17]. Despite the challenges, RNA-Seq of total RNA provides a unique opportunity to perform unbiased, genome-wide studies without a priori hypotheses and has the potential to identify novel RNA transcripts and isoforms [16]. With the falling costs of RNA-Seq (allowing samples to be sequenced to a much greater ‘depth’) and the development of specialist RNA library kits (enabling sequencing with smaller amounts of input RNA), it is now possible to interrogate the circulating, cell-free transcriptome in health and disease.

To date, approximately 65 lncRNAs have been associated with CAD or HF [10,11,12,15]. Of these, *H19* [18,19], *CoroMarker* [20], *SMILR* [21], *HOTAIR* [22] and *LIPCAR* [19,23] are notable examples owing to their presence in plasma or serum and their potential as diagnostic or prognostic markers for CAD or HF. Higher circulating levels of *H19*, *CoroMarker* and *LIPCAR* have been associated with the presence of CAD [18,19,20,23], and higher circulating levels of *LIPCAR* have also been associated with increased risk of cardiovascular mortality in patients with HF [23]. Higher circulating levels of *SMILR* have been associated with atherosclerotic plaque instability and inflammation [21]. In contrast, *HOTAIR* appears to be protective of cardiomyocytes, and lower circulating levels have been associated with acute myocardial infarction [22].

To extend the list of candidate biomarkers for CAD and HF, this study aimed to develop a short-read RNA-Seq protocol to detect mRNAs, lncRNAs and circRNAs (including putative novel lncRNAs) in human plasma. This protocol was applied to plasma from patients with established stable CAD (samples collected ~4 months after an acute coronary event) and healthy controls to screen for candidate mRNA, lncRNA and circRNA biomarkers associated with the presence of coronary artery disease and the progression from CAD to HF. We hypothesised that circulating levels of non-coding RNAs in patients with stable CAD may reflect progression of atherosclerotic disease or adverse myocardial remodeling and may help identify patients at risk of future cardiovascular events. Here, we demonstrate that mRNAs, lncRNAs and circRNAs can be reliably detected in human plasma by deep RNA-Seq, and we report novel candidate biomarkers for the presence of CAD.

## 2. Materials and Methods

### 2.1. Coronary Heart Disease Cohort Study (CDCS) 

From July 2002, patients (*n* = 2140) admitted to either Christchurch Hospital or Auckland City Hospital, New Zealand, were recruited into the Coronary Disease Cohort Study (CDCS) [24]. Inclusion criteria were ischaemic discomfort plus one or more of the following: ECG changes (ST-segment depression or elevation of at least 0.5 mm, T-wave inversion of at least 3 mm in at least 3 leads, or left bundle branch block), elevated levels of cardiac markers, a history of coronary disease or 64 years of age in patients with diabetes mellitus or vascular disease. Patients were excluded from the study if they had a severe comorbidity that limited their life expectancy to 3 years. Within the CDCS cohort, unstable angina accounted for 26.1% of all diagnoses at discharge, non-ST-segment elevation MI (NSTEMI) for 51.2% and ST-segment elevation MI (STEMI) for 22.7%. Anthropometric and clinical characteristics were recorded at planned follow-up clinic visits at baseline, 4 months and 12 months after admission. Clinical events were recorded from questionnaires, patient notes and NZHIS and hospital PMS databases. Median follow-up was 3.7 years (range, 0.1–7.9 years). The study conformed to the principles outlined in the Declaration of Helsinki and Title 45, US Code of Federal Regulations, Part 46, was approved by the New Zealand Multi-region Ethics Committee (Reference No. CTY/02/02/018) and registered with the Australian New Zealand Clinical Trials Registry (ACTRN12605000431628). Each participating patient provided written, informed consent. 

### 2.2. Canterbury Healthy Volunteers Cohort

Volunteers randomly selected from the Canterbury, New Zealand electoral rolls and age- and sex-matched to existing Christchurch Heart Institute acute coronary syndromes, MI and HF patient cohorts were recruited into the Canterbury Healthy Volunteers Cohort between 2003 and 2013 (*n* = 3358) [25]. Participants were aged 18 to 100 years and were screened before recruitment using hospital Patient Management Systems databases to confirm they had no documented personal history of overt cardiovascular disease, including CAD and MI. Participants attended a research clinic where they completed a study questionnaire on their medical history, smoking status, alcohol consumption, and self-reported physical activity. Height, weight, waist, and hip measurements were documented, blood pressure was recorded (seated, with duplicate readings at least 10 min apart), and a blood sample was taken for neurohormone and genetic analyses. Subsequent cardiovascular events during follow-up were identified through the New Zealand Health Information Services (NZHIS) database, with a median follow-up of 9 years. The study was approved by the Upper South A Ethics Committee (Reference No. CTY/01/05/062), and each participant provided written, informed consent. 

### 2.3. Plasma Collection

Blood was taken from an indwelling intravenous cannula placed 30 min prior to sampling, with the patient remaining semi-recumbent. Peripheral whole blood was collected into EDTA tubes and centrifuged within 30 min of collection at 4 °C. Plasma was stored at −80 °C. 

### 2.4. Sample Selection

Plasma samples collected from 61 CDCS patients ~4 months post-index coronary event once the patients were stable and early left ventricular remodelling was underway were selected for RNA-Seq analysis, along with plasma from 31 heart-healthy controls (Supplementary Figure S1). The CDCS group included all patients from the CDCS cohort who developed *de novo* HF within 3 years of their index hospital admission and had at least 5 mL plasma available (*n* = 30, HF+) and a matched group of patients who remained free of HF over a median 5.0 years follow-up (n = 31, HF−). HF− patients and controls (*n* = 31) were matched to the nearest HF+ group matched for age, sex, ethnicity, body mass index, smoking status and activity. In addition HF− patients were matched to the nearest HF+ group matched for heart rate, LVEF, E/e’, creatinine, previous medical history (hypertension, diabetes, myocardial infarction, valve disease, peripheral vascular disease, cerebrovascular accident, pulmonary disorder, coronary artery bypass grafting and percutaneous coronary intervention), severity of disease (plasma NT-proBNP, plasma high-sensitivity troponin I) and medications (beta blockers, angiotensin converting enzyme inhibitors, angiotensin-receptor blockers, statins and diuretics). Matching was performed with the MatchIt package (https://github.com/kosukeimai/MatchIt, accessed on 20 August 2018 [26]) in R [27]. 

### 2.5. Extraction of Circulating Cell-Free RNA from Plasma

Details of RNA extraction and quantitation are provided in the Supplementary Methods. Briefly, RNA extraction and clean-up from plasma were performed with Norgen Plasma/Serum RNA Purification kits (Norgen Biotek Corporation, Thorold, ON, Canada) according to the manufacturer’s instructions. This kit purifies RNA from up to 5 mL of fresh or frozen serum/plasma and concentrates high purity, cell-free circulating and exosomal RNA using a two-column method. RNA was quantified using the QubitTM adapted protocol [28] and stored at −80 °C prior to sequencing. 

### 2.6. Plasma RNA Sequencing

Details of total RNA-sequencing library preparation are provided in the Supplementary Methods. Briefly, RNA libraries were prepared using 8 μL input total RNA and the SMARTer^®^ Stranded Total RNA-Seq Kit v2–Pico Input Mammalian (Takara Bio, San Jose, CA, USA). All libraries were normalised and equimolarly pooled before being paired-end sequenced on an Illumina NovaSeq 6000 S1 flowcell using v1.5 chemistry, generating ~100 million reads per sample at 2 × 100 bp read length. Library preparation was performed by the Otago Genomics Facility (Dunedin, New Zealand), and sequencing was performed by the Ramaciotti Centre for Genomics (Sydney, Australia). 

### 2.7. Bioinformatics Pipeline

The bioinformatics pipeline was designed to detect mRNAs, lncRNAs and circRNAs and generate data on putative novel lncRNA and circRNA transcripts (summarised in Supplementary Figure S2) [29]. The pipeline is freely available to download at https://github.com/zoeward-nz/PhD, accessed on 20 August 2018. Briefly, libraries were de-multiplexed, adapter- and quality-trimmed using Trimmomatic [30] and quality assessed using Fastqc tools [31]. Resulting reads were aligned to the human reference genome (build GRCh38) using STAR2 [32], assembled into transcripts in StringTie [33], quantified using Salmon software [34] and aggregated to the gene or transcript level using the GENCODE.v33 annotation [35]. Gene/transcript expression was compared between groups using negative binomial generalised linear models with DESeq2 [36] (false discovery rate α < 0.01) as previously described [37]. For gene level analyses (annotated mRNA and lncRNA transcripts), only transcripts expressed at ≥1 transcript per million (TPM) in at least 90% of samples were considered to be robustly expressed and included in the analysis. To explore the possibility that our findings may have been confounded by differences in medications between patients and controls, normalised read counts were generated with the rlog function in DESeq2 [36] and associations re-tested for the top 20 transcripts, adjusting for treatment with ACE inhibitors, beta-blockers, statins or diuretics. Associations between the top 20 transcripts and selected clinical factors (including cardiac biomarkers) were assessed with Spearman correlation (after ln-transformation of data) and visualized on correlation matrices using the Corrplot package [38] in R [27]. CircExplorer2 [39] was used for circRNA detection, and the same detection threshold was applied to minimise detection of false-positive circRNAs (≥1 TPM in ≥90% of samples). To detect putative novel lncRNAs, filtering was relaxed to include low abundance transcripts with a read count ≥1 in at least 50% of samples. To minimise detection of false-positive novel lncRNAs, only multi-exonic transcripts aligning to the main chromosomes (not scaffold chromosomes) were included in the analysis. 

## 3. Results

### 3.1. Sequencing Quality Control and Patient Characteristics

RNA-Seq generated a median of 108 million reads per sample. Principal component analysis identified three outlying samples (one from each group, Supplementary Figure S3), which were removed. Two samples failed library preparation quality control owing to a low yield of cDNA; the third sample had a very low proportion of reads mapping to the human genome (2.5 million reads, 2.2%) of which approximately half aligned to non-coding regions of the genome, indicating a low amount input RNA contaminated with DNA. RNA-Seq mapping statistics for the remaining patients (HF− *n* = 30, HF+ *n* = 29, Controls n = 30) are summarised in Table 1 and shown in Supplementary Figure S4. Read depth varied considerably between samples, ranging from 84 to 193 million reads per sample but did not differ between patient and control groups (*p* = 0.666). The median number of uniquely aligned reads was 22 million reads per sample (19%), ranging from 3.4 to 64 million reads per sample (3.3–73.9%). Clinical characteristics of patients ~4 months after index admission (when patients were stable) and controls are shown in Table 2.

Plasma sample storage time was approximately 5 years longer for patients compared with controls (patients: median 14.2, range 10.6–16.9 years; controls: median 8.9, range 5.3–15.3 years, *p* < 0.001). While there was no correlation between sample storage time and total read depth (Spearman’s rho −0.03, *p* = 0.781), sample storage time was weakly negatively correlated with the number of uniquely mapped reads per sample (Spearman’s rho −0.353, *p* < 0.001), suggesting that RNA integrity in stored plasma declines over time. However, the number of uniquely aligned reads did not differ between patients and controls (patients: median 21.9, interquartile range 8.5–29.2 million reads per sample; controls: median 23.9, range 9.2–33.1 million reads per sample, *p* = 0.507), suggesting that the difference in sample storage time between patients and controls would be unlikely to adversely influence our findings.

### 3.2. Annotated mRNA and lncRNAs Associated with CAD and Progression to HF

**Figure 1:**
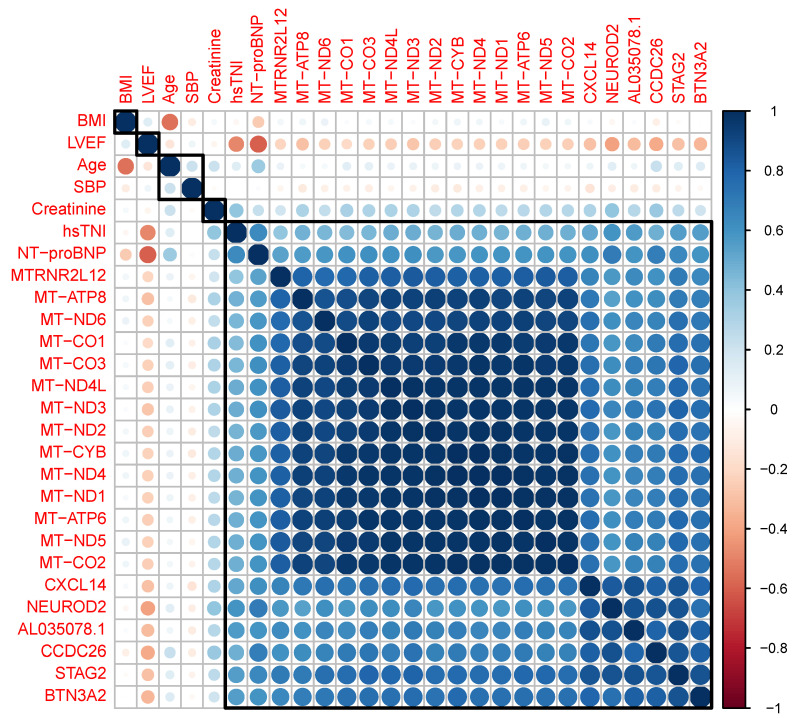
Correlation matrix showing the relationship between the 20 most differentially expressed transcripts and selected clinical factors in patients and controls. Hierarchical clustering indicated a strong correlation between transcripts, particularly those originating from the mitochondrial genome (i.e., all those prefaced by ‘MT’). All transcripts were moderately correlated with the cardiac biomarkers NT-proBNP and hsTNI, but not with age, BMI or SBP. The strength and direction of each association is indicated by circle size (larger circles indicate stronger correlations); blue indicates positive correlations; red indicates negative correlations: BMI, body mass index; hsTNI, high sensitivity troponin I; LVEF, left ventricular ejection fraction; NT-proBNP, amino-terminal pro B-type natriuretic peptide; SBP, systolic blood pressure.

Fragments from 4150 annotated genes were detected at ≥1 TPM reads in at least 90% of plasma samples (3986 mRNAs, 164 lncRNAs, GENCODE v33, Supplementary Table S1). We identified 170 transcripts (160 mRNAs, 10 lncRNAs) with altered levels in patients (HF+ and HF− groups combined) compared with controls (absolute fold change >1.2, *p* < 0.01 adjusted for multiple comparisons, Supplementary Table S1). The 20 most differentially expressed mRNA and lncRNA transcripts are shown in Table 3. Of these, 13 originated from the mitochondrial genome. The expression profiles of the mitochondrial transcripts were highly correlated with each other and moderately positively correlated with the cardiac markers NT-proBNP and hsTNI (Figure 1). 

Associations between the 20 most differentially expressed transcripts and CAD remained significant after adjustment for treatment with ACE inhibitors, beta-blockers, statins or diuretics, suggesting our findings were not confounded by drug treatments. All lncRNAs associated with higher expression in patients compared with controls (8 out of 10) overlapped (n = 7) or were in very close proximity (n = 1, 7308 bases) to CCCTC-binding factor (CTCF) transcription factor binding sites, suggesting they may have a potential gene regulatory role. No transcripts were found to be differentially expressed between HF+ and HF− groups.

### 3.3. Putative Novel lncRNAs Associated with CAD and Progression to HF

A total of 405 multi-exonic putative novel lncRNAs were detected in plasma (Supplementary Table S2). Putative lncRNAs were less abundant than annotated lncRNAs, with expression ranging from a median 0–50 TPM compared to 2–33,374 for annotated lncRNAs. Fragments from only two putative novel lncRNAs were detectable in all 89 samples. We identified two transcripts with higher plasma levels in patients compared with controls (MSTRG.752033.2 and MSTRG.76602.1 Table 4). No putative lncRNA transcripts were differentially expressed between HF+ and HF− groups.

### 3.4. circRNAs Associated with CAD and Progression to HF

We identified 227 putative circRNAs using CircExplorer2 [39] (Supplementary Table S3). There was no evidence that levels of any of the circRNAs differed between patients and controls at the nominal threshold (absolute fold change > 1.2, *p* < 0.01 after adjustment for multiple comparisons), although two circRNAs were borderline significant (*UBAC2* and *CLNS1A,* Table 5). None of the circRNA transcripts differed between HF+ and HF− groups.

## 4. Discussion

We report the first unbiased characterisation of the plasma transcriptome in patients with stable CAD. We show that mRNAs, lncRNAs and circRNAs can be reliably detected in human plasma by deep RNA-Seq and have identified multiple putative novel muti-exonic lncRNAs. Notably, the abundance of multiple transcripts was altered in association with CAD. Several of these transcripts originate from the mitochondrial genome and may be markers for underlying myocardial ischaemia and oxidative stress. If validated and confirmed to be altered in association with CAD in asymptomatic individuals (i.e., prior to an acute event), these transcripts could potentially be used as markers to help identify individuals at impending risk of an acute coronary event.

Sequencing total RNA from human plasma is technically challenging due to the low abundance of RNA, with only a few studies reported to date [8,9,16,17,40]. A major challenge in plasma RNA-Seq is that many sequencing reads represent degraded RNAs (i.e., transcripts are too short to map to a unique location in the human genome [16]) or bacterial transcripts owing to unavoidable contamination inherent in the sequencing kits [41,42,43]. Typically, 75–80% of reads cannot be aligned (‘mapped’) to the human reference genome [16] (median 81% in the current study) and are lost from the analysis, potentially leaving too few mapped reads to be informative. Here, we show it is possible to overcome this challenge through ultra-deep sequencing (~100 million reads per sample). This generated a median of 22 million uniquely mapped reads per sample, enabling robust detection of circulating cell-free RNAs. Further improvements could be achieved with enrichment procedures [44,45] and third-generation sequencing technologies that sequence RNA molecules directly [46] and avoid the need for PCR amplification (which may exacerbate the bacterial contamination problem). However, both these approaches have limitations. Enrichment may prohibit detection of novel transcripts, and third generation sequencing technologies currently require high amounts of input RNA that are typically not possible in plasma studies. We speculate that as RNA-Seq library kits improve, these technical hurdles will diminish and standardised methods for plasma RNA-Seq will evolve, enabling robust and reproducible analysis of the plasma transcriptome. 

Among the 20 mRNA transcripts most strongly associated with the presence of CAD, 13 originated from the mitochondrial genome. In patients with CAD, myocardial ischaemia and oxidative stress can promote mitochondrial apoptosis of cardiomyocytes, leading to release of damage-associated molecular patterns into the circulation, including cell-free mitochondrial RNA [47]. This is consistent with our observation that the majority of mitochondrial transcripts were more abundant in plasma from patients compared with controls. Notably, two mitochondrial transcripts, cytochrome C oxidase assembly factor 6 (COA6) [48] and NADH:ubiquinone oxidoreductase complex assembly factor 2 (NDUFAF2 [49]), had lower levels in patients compared with controls, which is consistent with previous reports linking their deficiency or absence to cardiomyopathies. Importantly, all mitochondrial RNAs were highly abundant in plasma, potentially making them ideal candidate biomarkers. Further work, in larger cohorts, will be needed to confirm these preliminary findings, ascertain the specificity of mitochondrial mRNAs for CAD and test whether one (or a combination of) mitochondrial mRNAs have the potential to identify people at impending risk of an adverse cardiovascular event. Our mRNA findings also support involvement of fibroblast growth factor 23 (FGF23) in CAD, which is expressed in the heart, promotes hypertrophy and remodelling and has been identified as an independent marker for cardiovascular risk in patients with dilated cardiomyopathy, ischaemic heart disease, acute decompensated and chronic HF [50,51,52,53,54], along with stromal antigen 2 (STAG2), which is required for proliferation and regulation of cardiac transcriptional programs [55]. 

Relatively few of the lncRNAs previously associated with CAD or HF (reviewed in [10,11,12,15]) have been measured in plasma or serum. Notable examples include *H19* [18,19], *CoroMarker* [20], *SMILR* [21], *HOTAIR* [22] and *LIPCAR* [19,23]. In our study, the abundance of *H19* fragments was 1.4-fold higher in patients than controls, and although this did not reach statistical significance, this fold-change is consistent with previous findings [18,19]. We were unable to detect fragments of *CoroMarker*, *SMILR*, *HOTAIR*, *LIPCAR* and other circulating lncRNAs previously associated with CAD, possibly due to their low abundance. 

In addition to *H19*, we detected fragments of five other lncRNAs previously associated with CAD and HF in peripheral blood mononucleocytes and human tissues (*RMRP* [56], *FTX* [57], *MALAT1* [58], *ZFAS1* [59,60] and *GAS5* [61,62,63]). In our study, *RMRP* was >2-fold lower in *RMRP* in plasma from patients compared with controls (*p* = 2.75 × 10^−5^) and ranked among the top 10 most differentially abundant lncRNAs. While our findings support a potential role for *RMRP* in stable CAD, the direction of association in our study contrasted to that previously observed in the human left ventricle, where patients with ischaemic heart failure had significantly higher expression of *RMRP* compared to controls [56]. The apparent mismatch between plasma and tissue might be explained by a reduction in the secretion of *RMRP* into plasma, leading to higher levels in tissue and lower levels in plasma compared to controls. Alternatively, lack of concordance between the two studies might also reflect differences in the pathology of CAD and HF or the timing of sample collection in the disease course. In contrast to *RMRP*, we did not detect differences in the abundance of the other lncRNAs previously associated with CAD or HF (*FTX*, *MALAT1*, *ZFAS1* and *GAS5*) between patients and controls. While these lncRNAs may have important roles in the pathophysiology of CAD and HF and may be potential therapeutic targets, our findings suggest they are unlikely to have strong potential as biomarkers for stable CAD. 

Notably, the majority of lncRNAs associated with the presence of CAD in our study overlapped or were in close proximity to CTCF binding sites, suggesting a potential regulatory function. CTCF is a transcription factor that, along with the cohesion complex, creates large loop domains within nuclear DNA called topologically associated domains. DNA contacts within each topologically associated domain are strong enabling enhancers and promoters to be brought into close contact to regulate gene expression [64]. Several lncRNAs have been shown to regulate CTCF [65,66,67,68]. Further analysis of these lncRNAs with techniques such as CLIP sequencing (where the RNA-protein complex is cross-linked and then immunoprecipitated, followed by RNA-Seq) are needed to explore whether the lncRNAs identified here may modulate gene expression and CAD risk through CTCF binding. Notably, all putative novel lncRNA transcripts were seen at a much lower abundance than the annotated lncRNAs, with only 2 novel lncRNAs detected in all 89 samples. Of the >400 putative novel lncRNAs present in >90% of samples, 2 were higher in patients compared with controls. While their low abundance means they may be unlikely to have clinical utility as markers for CAD (unless they were found to be extremely specific), selecting those transcripts that are most abundant, strongly differentially expressed and multi-exonic (less likely to be false positives due to the detection of the same splice junction in several samples) would be a way to prioritise novel lncRNAs for validation. 

To date, very few studies have investigated the plasma ‘circRNAome’ using RNA-Seq [69]. CircRNAs are potentially excellent biomarkers in biofluids as, due to their closed, circular structure, they are protected from degradation by RNases [6,70,71]. Despite not enriching for circRNAs, we robustly detected >200 circRNAs in human plasma. While no significantly differentially expressed circRNAs were identified using a conservative filter of *p*-adjusted <0.01, our finding that *circUBAC2* ranked as one of the most promising candidates supports a previous report that showed higher circulating levels of *circUBAC2* patients with myocardial infarction compared to healthy controls and, when combined with four other circRNAs, had good sensitivity and specificity for the diagnosis of myocardial infarction [72]. Our findings expand this work by suggesting that *circUBAC2* may also be elevated in patients with stable CAD. 

Our study has three major limitations. First, the relatively small sample size limited our power to detect differences in the circulating RNA profile between groups. Consequently, we may have missed some important associations with CAD, and we were unable to detect associations with subsequent HF. This may have been compounded by the close matching between patients who subsequently developed HF and those who did not. Second, we cannot completely exclude the possibility that our findings may be confounded by the effects of medications or sample storage time. Third, we have been unable to identify a large, well-characterised cohort of patients with blood sampling ~4 months after an acute coronary event in which to validate our findings. Consequently, further work is needed to confirm the specificity of our findings for CAD and establish their potential clinical utility. 

In summary, we have demonstrated that mRNAs, lncRNAs and circRNAs can be reliably detected in human plasma by deep RNA-Seq and have identified several promising candidate mRNA and lncRNA biomarkers for the presence of stable CAD. While RNA-Seq in human plasma is technically challenging due to the low abundance of RNA, this can be overcome by ultra-deep sequencing (>100 million paired reads per sample). In future, advances in library preparation kits, enrichment techniques and sequencing technologies will improve the detection of cell-free RNA in plasma and facilitate widespread use of RNA-Seq for the analysis of the plasma transcriptome.

## Figures and Tables

**Table 1:** RNA-Seq Mapping Statistics in Patients and Controls.

	Controls (*n* = 30)	HF− (*n* = 30)	HF+ (*n* = 29)
Total reads (M) *	111.2 (100.1–113.2)	101.9 (92.3–117.0)	110.2 (101.4–117.8)
Reads uniquely mapped (M) *	23.9 (9.2–33.1)	22.4 (7.6–28.7)	15.0 (9.1–30.3)
Reads uniquely mapped (%) *	18.9 (8.5–29.6)	19.0 (7.8–27.8)	13.6 (8.8–29.6)

* Median (interquartile range).

**Table 2:** Clinical characteristics of patients and controls.

	Controls (*n* = 30)	HF− (*n* = 30)	HF+ (*n* = 29)
Age (years) *	70 (60–77)	70 (63–76)	72 (60–77)
Male sex †	21 (70%)	22 (73%)	18 (62%)
European ethnicity †	26 (87%)	25 (83%)	21 (72%)
Cigarette smoker †	0 (0%)	0 (0%)	1 (3%)
SBP (mmHg) *	140 (130–149)	135 (121–160)	130 (111–143)
BMI (kg/m^2^) *	29 (25–33)	29 (26–33)	29 (25–34)
Diagnosis †	-	UA: 7 (23%)	UA: 6 (21%)
	-	NSTEMI: 15 (50%)	NSTEMI: 16 (55%)
	-	STEMI: 8 (27%)	STEMI: 7 (24%)
Type 2 Diabetes †	2 (7%)	11 (37%)	11 (38%)
Hypertension †	9 (30%)	22 (73%)	22 (76%)
Atrial fibrillation †	0 (0%)	5 (17%)	5 (17%)
Creatinine (μg/L) *	92 (78–99)	97 (85–109)	102 (99–116)
hsTnI (ng/L) *	2.9 (1.7–5.1)	8.0 (5.8–14.9)	11.1 (6.7–24.3)
NT-proBNP (pmol/L) *	8.0 (3.8–25.3)	92 (47–141)	146 (67–233)
LVEF (%) *	68 (63–74)	64 (52–75)	64 (52–75)
Beta blockers †	3 (10%)	27 (90%)	23 (79%)
ACE inhibitors or ARBs †	5 (17%)	19 (63%)	18 (62%)
Statins †	8 (27%)	27 (90%)	27 (93%)
Diuretics †	3 (10%)	8 (27%)	10 (35%)

* Median (interquartile range); † number of patients (%); ACE, angiotensin-converting enzyme; ARB, angiotensin II type I receptor blockers; BMI, body mass index; eGFR, estimated glomerular filtration rate; hsTnI, high sensitivity troponin I; NSTEMI, non ST-elevation myocardial infarction; NT-proBNP, amino-terminal pro B-type natriuretic peptide; SBP, systolic blood pressure; STEMI, ST-elevation myocardial infarction; UA, unstable angina.

**Table 3:** The most differentially abundant mRNA and lncRNA transcript fragments in plasma from patients compared with controls.

Gene Name	Transcript Type	Log 2 Fold Change (Standard Error)	*p*-Value *
STAG2	Protein coding	1.61 (0.12)	1.62 × 10^−36^
NEUROD2	Protein coding	1.89 (0.15)	1.48 × 10^−31^
MT-ND3	Protein coding	2.09 (0.18)	5.75 × 10^−29^
MT-ND5	Protein coding	2.13 (0.19)	5.80 × 10^−28^
MT-CO2	Protein coding	2.08 (0.18)	1.00 × 10^−27^
MT-ND6	Protein coding	2.22 (0.20)	1.64 × 10^−27^
MT-CYB	Protein coding	2.10 (0.19)	3.29 × 10^−27^
MT-ND1	Protein coding	2.16 (0.19)	1.65 × 10^−26^
MT-ATP6	Protein coding	2.00 (0.18)	3.33 × 10^−26^
MT-ND4L	Protein coding	1.98 (0.18)	5.77 × 10^−26^
AL035078.1	lncRNA	1.77 (0.16)	6.48 × 10^−26^
CXCL14	Protein coding	1.98 (0.18)	1.40 × 10^−25^
MT-CO1	Protein coding	2.04 (0.19)	1.84 × 10^−25^
CCDC26	lncRNA	1.72 (0.16)	4.51 × 10^−25^
MT-ND2	Protein coding	1.98 (0.18)	6.49 × 10^−25^
MT-ND4	Protein coding	2.01 (0.19)	9.42 × 10^−25^
MT-CO3	Protein coding	2.01 (0.19)	1.82 × 10^−24^
BTN3A2	Protein coding	1.39 (0.13)	6.75 × 10^−24^
MT-ATP8	Protein coding	2.05 (0.20)	3.25 × 10^−22^
MTRNR2L12	Protein coding	2.13 (0.22)	8.15 × 10^−20^

* *p*-value adjusted for multiple comparisons.

**Table 4:** The most differentially abundant putative novel lncRNA transcript fragments in plasma from patients compared with controls.

Gene Name	Chromosomal Position (Strand)	Log 2 Fold Change (Standard Error)	*p*-Value *
MSTRG.752033.2 MSTRG.76602.1	7: 113240035−113240441 10: 3408557−3409012	1.69 (0.40) 2.44 (0.60)	1.66 × 10^−3^ 3.36 × 10^−3^

* *p*-value adjusted for multiple comparisons.

**Table 5:** The most differentially abundant circRNA transcript fragments in plasma from patients compared with controls.

Gene Name	Chromosomal Position (Strand)	Log 2 Fold Change (Standard Error)	*p*-Value *
UBCA2	13:99238426−99244624 (+)	0.43 (0.16)	4.70 × 10^−2^
CLNS1A	11:77619605−77625818 (-)	0.74 (0.28)	6.50 × 10^−2^

* *p*-value adjusted for multiple comparisons.

## Data Availability

RNA-seq data have been deposited in the National Center for Biotechnology Information Gene Expression Omnibus database (www.ncbi.nlm.nih.gov/geo, accessed on 12 June 2022; Accession No. GSE208194). The bioinformatic pipeline is freely available to download at https://github.com/zoeward-nz/PhD, accessed on 12 June 2022. Clinical data will be made available in accordance with our ethics approvals, upon reasonable request.

## Supplementary Materials

The following supporting information can be downloaded at: https://www.mdpi.com/article/10.3390/cells11203191/s1, Supplementary Methods; Figure S1: Selection of plasma samples from CDCS and Control cohorts; Figure S2: Schematic of bioinformatic pipeline; Figure S3: Principal component analysis of the normalised read counts for each sample; Figure S4: The percentage and number (millions) of reads aligning to the human genome by group (outlier samples removed); Table S1: Annotated mRNA and lncRNAs in plasma and associations with CAD; Table S2: CircRNAs in plasma and associations with CAD; Table S3: Putative novel lncRNAs in plasma and associations with CAD.
