# Supplementary material for: Identifying Candidate Circulating RNA Markers for Coronary Artery Disease by Deep RNA-Sequencing in Human Plasma
Source: Cells. 2022 Oct 11;11(20):3191. doi: 10.3390/cells11203191 (PMC9599983; doi:10.3390/cells11203191)
Supplement: Supplementary file 1 [file cells-11-03191-s001.zip › Supplementary Methods and Figures.pdf]

Supplementary Figures

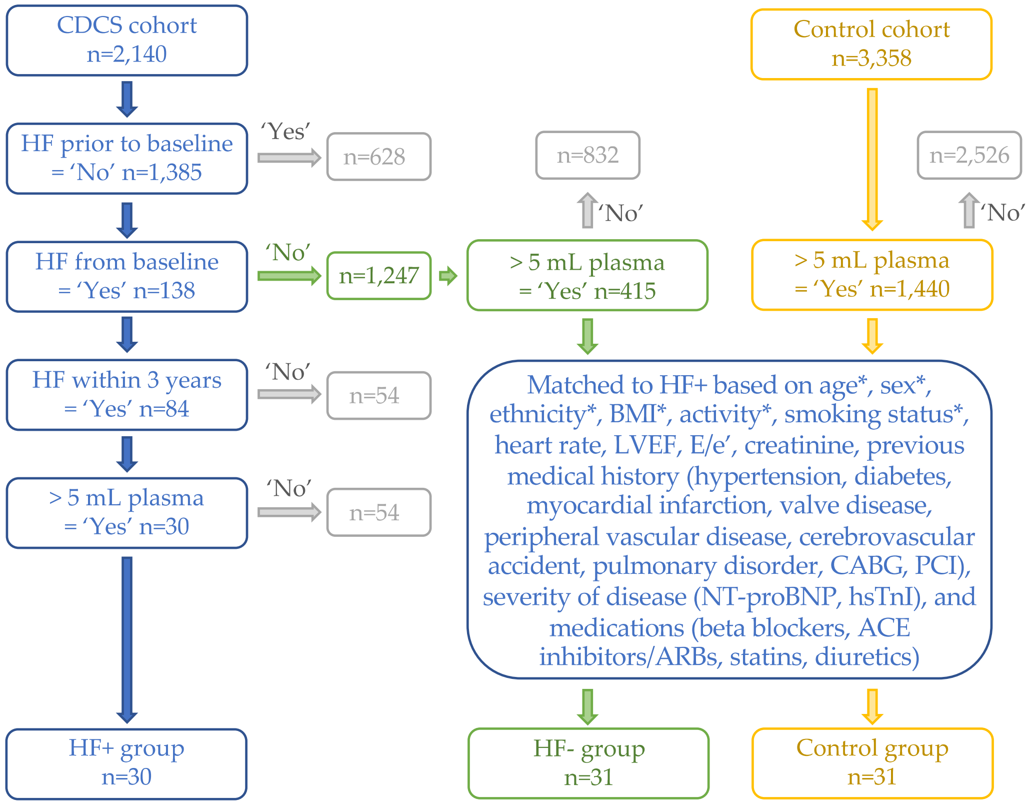

Supplementary Figure S1. Selection of plasma samples from CDCS and Control cohorts. Flow diagram showing selection of patients with a *de novo* diagnosis of HF within 3 years of admission for an acute coronary syndrome and a minimum of 5mL plasma available at ~4-month timepoint (HF+, n=30). HF- samples (n=31) and Controls (n=31) were matched to HF+ positive samples using nearest matching with the MatchIt package in R (HF- matched on all variables, Controls matched on variables indicated with *). ACE, angiotensin-converting enzyme; ARB, angiotensin II type I receptor blockers; BMI, body mass index; CABG, coronary artery bypass graft; HF, heart failure; hsTnI, high sensitivity troponin I; LVEF, left ventricular ejection fraction; NT-proBNP, amino-terminal pro B-type natriuretic peptide; PCI, percutaneous coronary intervention.

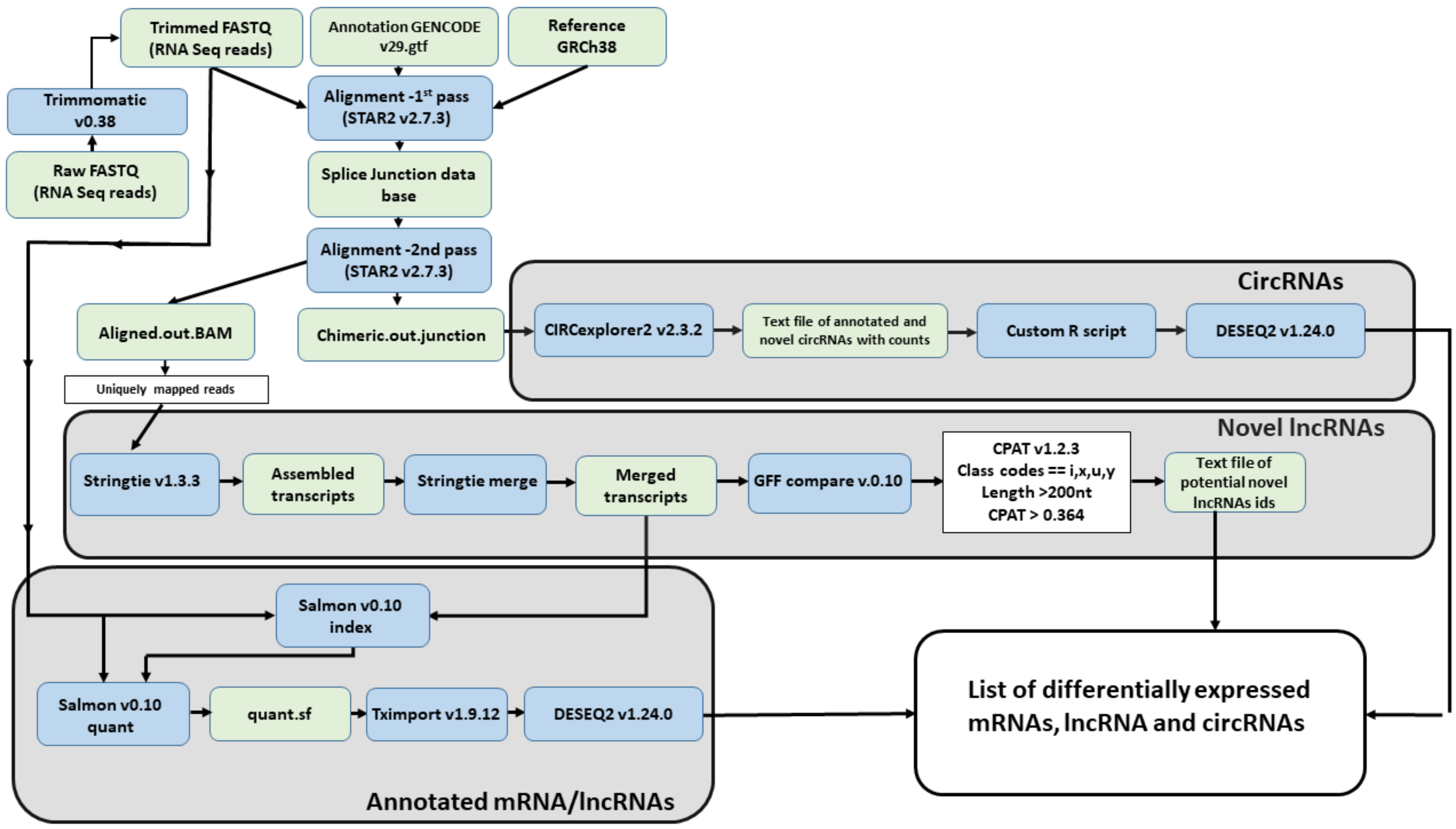

Supplementary Figure S2. Schematic of bioinformatic pipeline. Blue boxes indicate software packages, green boxes represent the input or output files. The pipeline is freely available to download at https://github.com/zoeward-nz/PhD.

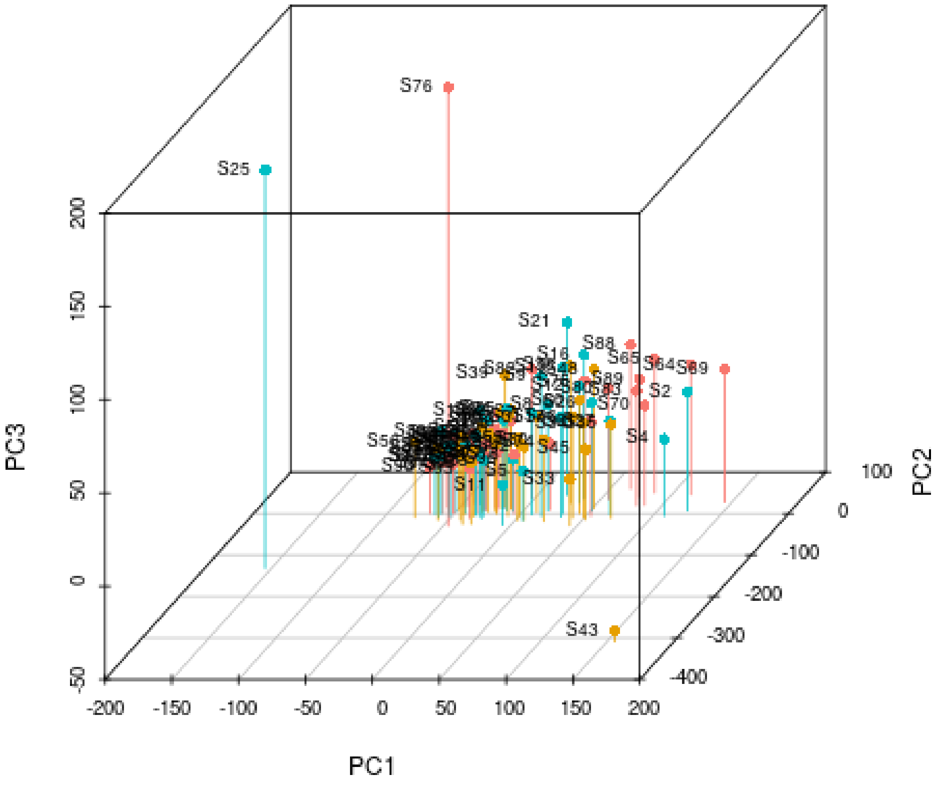

Supplementary Figure S3. Principal component analysis of the normalised read counts for each sample. Samples S25, S76 and S43 were identified as outlying samples and removed from the analysis. S25 and S76 were flagged by the sequencing facility as failing the minimum cDNA yield during library quality control. S43 had the smallest number of reads aligning to the genome and strong evidence of DNA contamination (approximately 50% of reads aligning to non-coding regions).

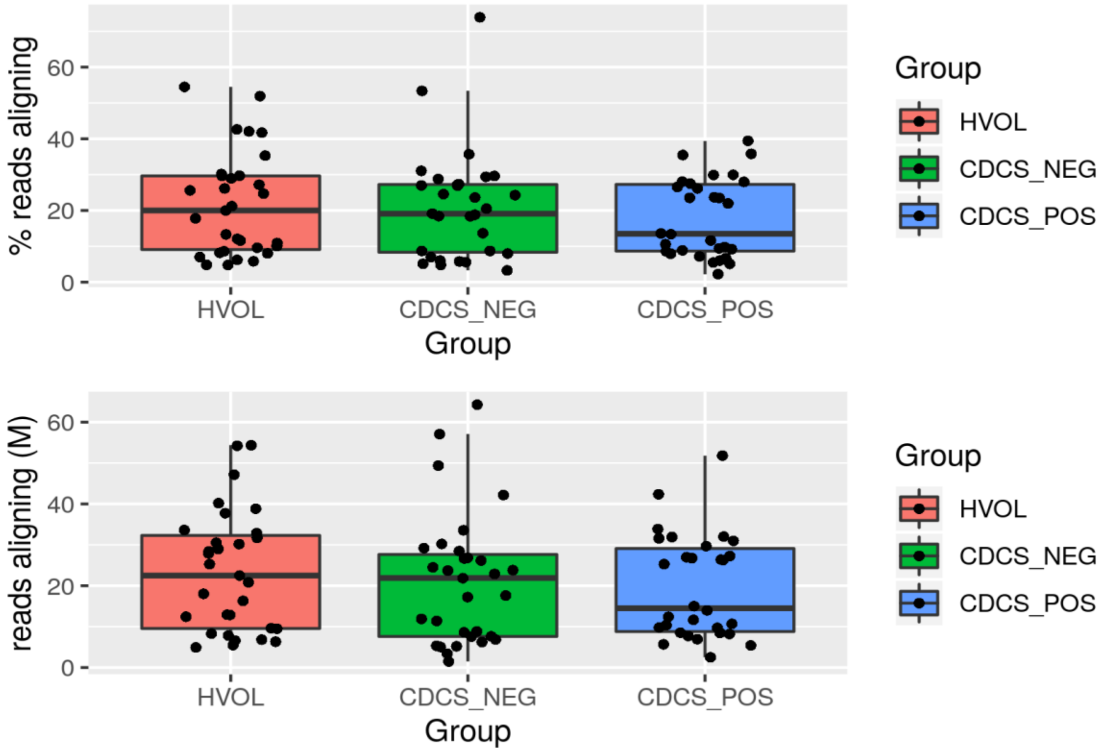

Supplementary Figure S4. The percentage and number (millions) of reads aligning to the human genome by group (outlier samples removed). Box and whisker plots indicating a similar number of reads uniquely mapped to the genome across the groups. HVOL, Control cohort (n=30); CDCS_NEG, HF- (n=30); CDCS_POS: HF+ (n=29).

Supplementary Methods

RNA extraction

Following selection of plasma samples from CDCS and HVOLs cohorts (HVOL n=31, CDCS HF – n=31, CDCS HF + n=30), I performed RNA extraction and clean-up from plasma using Norgen Plasma/Serum RNA Purification kits (cat #56200, Norgen Biotek Corporation, Thorold, Canada). This kit purifies RNA from up to 5 mL of fresh or frozen serum/plasma and concentrates high purity, cell-free circulating and exosomal RNA using a two-column method. The first column processes the large volume of serum/plasma fluid which is followed by a concentration of the RNA on a second mini column. Frozen plasma was thawed on ice and centrifuged at 400g for 2 minutes to remove cell debris. Up to 5mL of plasma was transferred to a 50mL tube (if the sample volume was less than 5mL it was topped up to 5mL using nuclease free water). Lysis Buffer A (15mL) along with 150µl of β-mercaptoethanol was added to each plasma sample and vortexed for 10 seconds. Isopropanol (10mL) was added and vortexed for 10 seconds. Then 15mL of this solution was transferred to a Maxi Spin column with collection tube (lids were left loose) and centrifuged for 3 minutes at 1000g, to bind the RNA. The flow-through was discarded, and column and tube reassembled. This was repeated until all of the mixture had been added to the column. Wash Solution A (5mL) was added and centrifuged for 3 minutes at 1,000g. The flow-through was discarded, and column and tube reassembled, and this wash was repeated a second time. The column was spun empty at 2000g for 3 minutes to dry the column. The Maxi Spin column was transferred to a fresh 50mL tube, 800µl of Elution Buffer F was added to the column, incubated at room temperature for 2 minutes and then centrifuged for 2 minutes at 500g. To maximise RNA recovery the eluted RNA was reloaded onto the same Maxi Spin column, incubated at room temperature for 2 minutes and then centrifuged for 2 minutes at 500g. To concentrate the RNA, 600µl of Lysis Buffer A was added to the eluate and vortexed for 10 seconds. This was followed by adding 800 µl of 96-100% Ethanol and vortexing for 10 seconds, and then 750µl of the ethanol-RNA eluate mix was transferred to a Mini Spin column and centrifuged for 2 minutes at 3,300g. The flow-through was discarded and this step was repeated until all of the mixture had been transferred. For every sample, a mix of 15 µL of DNase I and 100 µL of Enzyme Incubation Buffer was prepared using Norgen's RNase-Free DNase I Kit (Product # 25710). Prior to DNase I treatment, 400µl of Wash Solution A was added to the column, centrifuged for 3 minutes at 3,300g and the flow-through discarded. After adding 100µl of the prepared RNase-free DNase I solution to the column and centrifuging at 8,000g for 1 minute, the flow-through was pipetted back to the column and incubated at 25-30°C for 15 minutes, to ensure maximal degradation of DNA. To wash the RNA bound to the column, 400µl of Wash Solution A was added to the column, centrifuged for 3 minutes at 3,300g and the flow- through discarded. This step was repeated a second time to ensure removal of digested DNA. The column was spun empty for 2 minutes at 13,000g to dry it completely before transferring it to a fresh 1.7mL Elution tube and adding 50µL of Elution Solution A to the column, incubating at room temperature for 2 minutes and centrifuging for 1 minute at 400g followed by 2 minutes at 5,800g. To maximise RNA recovery the eluted buffer was pipetted back to the column, incubated at room temperature for a further 2 minutes and centrifuged for 1 minute at 400g followed by 2 minutes at 5,800g. RNA was quantified using the QubitTM adapted protocol (Section 3.3.3.1.3). This final 50µl elution containing the RNA was stored at -80C prior to sequencing.

Fluorometry (QubitTM) Adapted protocol of RNA quantification for plasma

Because the concentration of RNA extracted from human plasma was below the detection limit of the QubitTM high sensitivity RNA kit described above, a modified protocol that could detect RNA concentrations between 250pg/µL and 55.6pg/µL [365] was used. The QubitTM working solution and standards were prepared as described above. A 2.5ng/µL RNA 'spike in' master mix was prepared by diluting the QubitTM RNA Standard #2 4 -fold with RNase free water. The 'spike in' master mix

(182μL) was mixed with 18μL of RNAse free water ('spike in' alone tube) or 17μL of RNAse free water and 1μL of sample. All tubes were vortexed for 3 seconds, centrifuged for ~ 5 seconds (to collect the solution at the bottom of the tube) and incubated at room temperature for 2 minutes. After calibrating the QubitTM fluorometer with both standards (described above) the RNA 'spike in' alone tube was measured (Read 1) followed by the RNA sample tubes (Read 2). RNA sample concentration was calculated as:

Concentration (pg/uL) = (Read2 – Read1) (pg/μL) × 200 (μL) ÷ volume of sample added (μL)

Preparation of Total RNA Libraries

Total RNA-sequencing libraries were prepared with the SMARTer® Stranded Total RNA-Seq Kit v2 - Pico Input Mammalian (Takara Bio, USA) by the Otago Genomics Facility (Dunedin, New Zealand). Details are provided below.

First strand synthesis

Option 2 (starting with degraded RNA) without fragmentation was used. RNA sample (8μl) was mixed with 1μl of SMART Pico Oligos Mix v2 on ice and incubated at 72°C for 3 minutes, then chilled rack for 2 minutes. The First-Strand Master Mix was prepared with 4μl 5x First-Strand Buffer, 4.5μl SMART TSO Mix v2, 0.5μl RNase Inhibitor and 2μl SMARTScribe Reverse Transcriptase, added to each reaction tube and vortexed and incubated at 42°C for 90 minutes, 70°C for 10 minutes and then held at 4°C

Addition of Illumina adapters and indexes

A PCR master mix with 2μL nuclease-free water, 25 μl SeqAmp CB PCR Buffer (2X), 1μl SeqAmp DNA Polymerase was added to each sample. PCR Primer HT was added (1μl), and PCR was performed: 94°C for 1 minute, 5 cycles of 98°C for 15 seconds, 55°C for 15 seconds, 68°C for 30 seconds, 68°C for 2 minutes.

Purification of the RNA-Seq Library Using AMPure Beads

AMPure beads (40μL/sample), were added to each sample tube and incubated for 8 minutes. Tubes were placed onto a magnetic separation device for 5 minutes, the supernatant was removed and discarded and 200μl of freshly made 80% ethanol was added then removed and discarded, and the wash step was repeated. Pellets were air dried, 52μl of nuclease-free water added, incubated for 5 minutes at room temperature, and placed back onto the magnetic separation device for 1 minute. Supernatant (50μl) was transferred to a new tube, mixed with 40μL of AMPure beads and incubated for 8 minutes.

Depletion of Ribosomal cDNA with ZapR v2 and R-Probes v2

Tubes were placed on the magnetic separation device for 5 minutes, supernatant removed and discarded, and 200 μl of freshly made 80% ethanol added to each sample. Supernatant was removed and discarded, and the wash step was repeated. Tubes were aired dried on the magnetic separation device. A ZapR master mix was prepared: 16.8 μl Nuclease-Free Water, 2.2 μl 10X ZapR Buffer, 1.5 μl ZapR v2 and lastly 1.5 μl 'activated' R-Probes v2 (that bind rRNA and mitochondrial rRNA which had been preheated hot-lid thermal cycler at 72°C for 2 minutes and held at 4°C for at least 2 minutes). The dried beads were resuspended in 22 μl of the ZapR master mix, incubated at room temperature for 5 minutes and placed on the magnetic separation device for 1 minute. Supernatant was removed and transferred to a new PCR tube. Tubes were incubated in a preheated hot-lid thermal cycler at 37°C for 60 minutes followed by 72°C for 10 minutes then held at 4°C.

Final RNA-Seq Library Amplification

Library fragments were further enriched in a second round of PCR. A PCR master mix was prepared: 26 μl Nuclease-Free Water, 50 μl SeqAmp CB PCR Buffer, 2 μl PCR2 Primers v2 and 2 μl SeqAmp DNA

Polymerase. 80µl of master mix was added to each sample tube. Tubes were incubated for 94°C for 1 minute, 16 cycles of 98°C for 15 seconds, 55°C for 15 seconds, 68°C for 30 seconds, and held at 4°C.

Purification of Final RNA-Seq Library Using AMPure Beads

A second purification step with AMPure beads. Essentially the process was the same as Section 3.5.3 but instead of 40µl/sample of AMPure beads, 100µl AMPure beads were added to each sample. Also, the final elution was into 12µl of Tris buffer. The samples were then sent to the Ramaciotti Centre for Genomics, Sydney, Australia for sequencing on the NovaSeq6000.
